# Hybrid Clustering of Single-Cell Gene Expression and Spatial Information *via* Integrated NMF and K-Means

**DOI:** 10.3389/fgene.2021.763263

**Published:** 2021-11-08

**Authors:** Sooyoun Oh, Haesun Park, Xiuwei Zhang

**Affiliations:** School of Computational Science and Engineering, Georgia Institute of Technology, Atlanta, GA, United States

**Keywords:** single cell transcriptomics, spatial locations, cell identity, non-negative matrix factorization, data integration

## Abstract

Advances in single cell transcriptomics have allowed us to study the identity of single cells. This has led to the discovery of new cell types and high resolution tissue maps of them. Technologies that measure multiple modalities of such data add more detail, but they also complicate data integration. We offer an integrated analysis of the spatial location and gene expression profiles of cells to determine their identity. We propose scHybridNMF (single-cell Hybrid Nonnegative Matrix Factorization), which performs cell type identification by combining sparse nonnegative matrix factorization (sparse NMF) with k-means clustering to cluster high-dimensional gene expression and low-dimensional location data. We show that, under multiple scenarios, including the cases where there is a small number of genes profiled and the location data is noisy, scHybridNMF outperforms sparse NMF, k-means, and an existing method that uses a hidden Markov random field to encode cell location and gene expression data for cell type identification.

## 1 Introduction

Advances in single cell RNA-Sequencing (scRNA-Seq) technology provided an unprecedented opportunity for researchers to study the identity and mechanisms of single cells (Morris, 2019). While scRNA-Seq data is a major type of data used to study single cells, it cannot fully determine the identity of a cell (McKinley et al., 2020). As such, it is important to consider other modalities such as chromatin accessibility (Cusanovich et al., 2015), protein abundance (Peterson et al., 2017), or spatial locations (Ståhl et al., 2016; Wang et al., 2018) of single cells. In particular, spatial location data can provide important information on the cells’ micro-environment and cell-cell interactions (Mayr et al., 2019). In certain tissues like the brain, cells at nearby locations tend to have the same type—daughter cells tend to keep the same type and location as their mother.

Technologies that jointly profile the location and gene expression of cells are often forced to measure a small set of genes (Zhu et al., 2018). Since clustering cells using smaller gene expression profiles can be inaccurate, incorporating the cell location data can improve its accuracy. However, reconciling single cell gene expression and location data for cell type identification is challenging because different data types can have differing scales, distributions, and types of noise (Efremova and Teichmann, 2020).

Computational methods that integrate multimodal data are crucial for learning a comprehensive picture of inter- and intra-cell processes (Efremova and Teichmann, 2020; Stuart and Satija, 2019). Promising nonnegative matrix factorization (NMF) models have been developed for cell type identification for multiple types or modalities of data (Shao and Höfer, 2017; Duren et al., 2018; Kotliar et al., 2019; Welch et al., 2019; Jin et al., 2020). However, none of these methods incorporate cell locations. On the other hand, Zhu et al. (2018) developed a HMRF (Hidden Markov Random Field) model and showed that the spatial location of cells can contribute to cell type identification.

We introduce a matrix low-rank approximation scheme, scHybridNMF (single-cell Hybrid NMF), to perform cell clustering by jointly processing cell location and gene expression data. We use a matrix low-rank approximation scheme because of the ease of preserving data characteristics through constraints and optimization terms. We combine sparse NMF with k-means clustering to cluster high-dimensional gene expression data and low-dimensional location data in an integrative way. We compare the performances of scHybridNMF against sparse NMF, k-means clustering, and HMRF on simulated and two real datasets, STARmap (Wang et al., 2018) and seqFISH+ (Eng et al., 2019), which both profile the mouse brain cortex.

## 2 Materials and Methods

Matrix low-rank approximations approximate matrices as products of lower-rank matrices. Many biological clustering frameworks are designed as matrix low-rank approximation schemes because they can easily incorporate prior biological knowledge and data constraints. We formulated scHybridNMF as a combination of multiple low-rank approximations. This formulation guided the gene expression-based cell clustering with cell location information. We chose sparse NMF and k-means clustering because they could be formulated as matrix low-rank approximations, and incorporating these methods was intuitive.

### 2.1 Review of Sparse Nonnegative Matrix Factorization and K-Means Clustering

K-means clustering is an unsupervised learning algorithm that clusters data points by comparing pairwise distances. This metric naturally pairs with location-based data because it determines the similarity between points by how physically close they are. Eq. 1 shows the matrix formulation for a Euclidean distance-based k-means objective for clustering $L\in R^{2\times n}$ , which represents location data.

$$\underset{\begin{array}{c} H_{L}\in {\left\{0,1\right\}}^{k\times n}\\ H_{L}^{T}1_{k}=1_{n} \end{array}}{\min}{\left\|L-W_{L}H_{L}\right\|}_{F}^{2},$$ (1)

where **1**_*k*_ and **1**_*n*_ are *k*- and *n*-length vectors of ones. The columns of $W_{L}\in R^{2\times k}$ contain *k* cluster centroids, and the columns of $H_{L}\in R^{k\times n}$ contain each point’s cluster membership. If a point *i* belongs to a cluster *j*, *H*_*L*_ (*j*, *i*) = 1 and *H*_*L*_ (*l*, *i*) = 0 for *l* ≠ *j*. The constraints preserve the hard-clustering requirement of k-means, as each data point can only belong to one cluster. This is equivalent to having one 1 per column of *H*_*L*_. Additionally, k-means does not require any pre-processing, such as building a location-based neighborhood graph, on location data. Pre-processing location data may remove many of their underlying characteristics.

NMF is a dimension reduction algorithm that is well-suited for high-dimensional data. Given a nonnegative input matrix $A\in {R\;}_{+}^{m\times n}$ , NMF computes two nonnegative factors, *H*_*A*_ and *W*_*A*_ of a specified reduced dimension size *k*, where *k* is generally much smaller than *m* and *n*. The columns of $W_{A}\in {R\;}_{+}^{m\times k}$ contain *k* cluster representatives, and the columns of $H_{A}\in {R\;}_{+}^{k\times n}$ contain cluster membership information.

Sparse NMF constrains the sparsity in each column of *H*_*A*_ (Kim and Park, 2007). It converts the soft clustering of NMF into more of a hard clustering—a data point will have fewer nonzero entries in the cluster membership matrix and be represented by fewer cluster representatives. Sparse NMF may be interpreted as a hard clustering method if we assign each data point to the cluster of the maximal element in its column of *H*_*A*_. For example, if the largest element in the first column of *H*_*A*_ is in the second entry, we can interpret the first data point as belonging to the second cluster.

Eq. 2 contains the formulation for sparse NMF. The first term is the objective term for standard NMF, which minimizes the difference between *A* and *W*_*A*_ *H*_*A*_. The low-rank factors from NMF are not inherently unique, so we normalize the columns of the computed *W*_*A*_ and scale the rows of *H*_*A*_ accordingly. The second term limits the size of the elements in *W*_*A*_, and the final term promotes the sparsity in each column of *H*_*A*_.

$$\underset{\left\{W_{A},H_{A}\right\}\geq 0}{\min}{\left\|A-W_{A}H_{A}\right\|}_{F}^{2}+\beta \|W_{A}{\|}_{F}^{2}+\gamma \overset{n}{\underset{i=1}{\sum }}\|H_{A}\left(:,i\right){\|}_{1}^{2}.$$ (2)

### 2.2 Multimodal Objective

Let $A\in {R\;}_{+}^{m\times n}$ denote the normalized gene expression matrix and $L\in R^{2\times n}$ denote the two-dimensional cell location coordinates, where *m* is the number of genes and *n* is the number of cells. To get the normalized gene expression matrix, we first scaled the rows of the raw count matrix, $\tilde{A}$ , by its library size, then set $A=\log_{2}\left(\tilde{A}+1\right)$ . We computed *W*_*A*_ and *H*_*A*_ from sparse NMF on the gene expression data and *W*_*L*_ and *H*_*L*_ from k-means clustering on the location data. We used the same *k* in both methods, which allowed for a direct comparison between the two data types. We assumed that *k* is already known for each dataset. Eq. 3 is the objective function for the multimodal clustering:

$$\underset{\left\{W_{A},H_{A}\right\}\geq 0}{\min}g\left(W_{A},H_{A}\right)=\underset{\left\{W_{A},H_{A}\right\}\geq 0}{\min}{\left\|A-W_{A}H_{A}\right\|}_{F}^{2}+\alpha {\left\|H_{A}-H_{A}\circ {\hat{H}}_{L}\right\|}_{F}^{2}.$$ (3)

In Eq. 3, ◦ represents the element-wise product between two matrices, and the second term forms the consensus between the clustering results from sparse NMF and k-means clustering. ${\hat{H}}_{L}$ was obtained by converting *H*_*L*_ into a matrix of confidence scores that considered how close each cell was to the edge of its location-based cluster. We found the index of two closest cluster centroids to each cell *i*, then assigned values to entries in ${\hat{H}}_{L}$ (Eq. 4). All other entries of ${\hat{H}}_{L}$ remained zero. As such, we compared *H*_*A*_ with ${\hat{H}}_{L}$ , and not with *H*_*L*_ directly.

$${\hat{H}}_{L}\left(j,i\right)=\left\{\begin{array}{cc} \frac{{\left\|W_{L}\left(:,j\right)-L\left(:,i\right)\right\|}_{2}}{\sum_{j^{\prime }=1}^{2}{\left\|W_{L}\left(:,j^{\prime }\right)-L\left(:,i\right)\right\|}_{2}},\;\; & \text{if }j\text{ is one of the top }2\text{ cluster indices for cell }i.\\ 0,\;\; & j\text{ for all other clusters}. \end{array}\right.$$ (4)

Instead of forcing *H*_*A*_ and ${\hat{H}}_{L}$ to be similar overall, the second term in Eq. 3 forced *H*_*A*_ and ${\hat{H}}_{L}$ to be similar in terms of their cluster memberships. In other words, the second term of Eq. 3 aimed to match the location of the largest element in each column of *H*_*A*_ and the location of the two nonzero elements in the corresponding column of ${\hat{H}}_{L}$ .

The main focus of this work was to use cell location information to aid the gene expression-based clustering of cells. Because we specifically adapted gene clusters to incorporate location cluster information, our design sought to align the cluster membership matrices while still considering the accuracy of the gene expression clustering. We did not include a sparsity term for *H*_*A*_, the final cluster membership matrix, because imposing the sparsity terms may eliminate nuance in the integration of both clustering schemes, and thus result in a loss of information that could better serve to cluster the cells.

### 2.3 Proposed Algorithm

scHybridNMF optimized Eq. 3 to combine the clusters of sparse NMF on *A* and k-means on *L*. To get the initial *H*_*A*_ for the consolidated algorithm, we ran sparse NMF on *A*. We then computed k-means clustering on *L*. We computed initial centroids by taking the means of each cell’s locations within the gene expression-based clusters.

scHybridNMF used block coordinate descent for computing *H*_*A*_ and *W*_*A*_. These two terms were computed via an alternating nonnegative least squares (ANLS) formulation.

$${\left\|H_{A}-H_{A}\circ {\hat{H}}_{L}\right\|}_{F}^{2}={\left\|H_{A}\circ 1_{k\times n}-H_{A}\circ {\hat{H}}_{L}\right\|}_{F}^{2}={\left\|H_{A}\circ C\right\|}_{F}^{2},$$ (5)

where $C=1_{k\times n}-{\hat{H}}_{L}$ and **1**_*k*×*n*_ is a *k* × *n* matrix of ones. We represented the element-wise product in a block-ANLS formulation by computing it column-by-column. Column *i* of *H*_*A*_ is updated as follows:

$$H_{A}\left(:,i\right)\leftarrow \underset{H_{A}\left(:,i\right)\geq 0}{\arg\;\min}{\left\|\left(\begin{array}{c} W_{A}\\ \sqrt{\alpha }*\mathrm{diag}\left(C\left(:,i\right)\right) \end{array}\right)H_{A}\left(:,i\right)-\left(\begin{array}{c} A\left(:,i\right)\\ 0_{k} \end{array}\right)\right\|}_{F}^{2},$$ (6)

where *i* ∈ {1, … , *k*}, **1**_*k*_ is a *k*-length vector of ones, and **0**_*k*_ is a *k*-length vector of zeros. Each column in *H*_*A*_ was element-wise multiplied to each column in *C* in Eq. 5, which can be represented as a left-multiplication of the column of *H*_*A*_ by a matrix whose diagonal entries are the corresponding column of *C*.

For *W*_*A*_, we used the following update rule:

$$W_{A}\leftarrow \underset{W_{A}\geq 0}{\arg\;\min}{\left\|A-W_{A}H_{A}\right\|}_{F}^{2}.$$ (7)

The overall scheme is described in Algorithm 1. There exist many stopping criteria that can be used. We used two: a maximum number of iterations and a normalized KKT condition residual check, as used in SymNMF (Kuang et al., 2015).

Algorithm 1 scHybridNMF: an algorithm to minimize Eq. 3

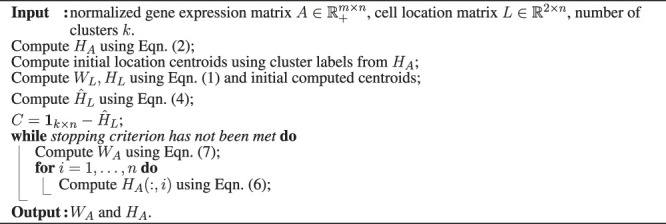

### 2.4 Parameters

In line 1 of Algorithm 1, we computed sparse NMF on the data matrix *A* through Eq. 2. This formulation involved *β* and *γ*, which controlled the size of the entries of *W*_*A*_ and the sparsity of *H*_*A*_, respectively. To ensure that the last two terms were proportionate to the first term in the formulation, we formulated *β* and *γ* to have a denominator of ${\left\|A\right\|}_{F}^{2}$ , which is the maximum value the first term can take. We also formulated the parameters based on the dimensions of *W*_*A*_ and *H*_*A*_. We set the numerator of *β* to be *m*, which is the number of rows of *W*_*A*_, and we set the numerator of *γ* to be *n*, which is the number of columns of *H*_*A*_. The final formulations were $\beta =\frac{m}{{\left\|A\right\|}_{F}^{2}}$ and $\gamma =\frac{n}{{\left\|A\right\|}_{F}^{2}}$ .

The parameter *α* in the hybrid clustering scheme was designed to control the degree to which the consensus clustering was influenced by the location-based clusters. The maximum number of iterations to run the main BCD was set to be 500 so it is not triggered as much as the other stopping criterion. The tolerance level, *tol*, of the normalized KKT residual check had a default value of 0.01. The relationship between *α* and *tol* is interesting. A smaller *α*, which prioritizes gene expression-based clusters, required a larger *tol*, as scHybridNMF’s clusters did not converge otherwise. Likewise, a larger *α*, which prioritizes cell location-based clusters, required a smaller *tol* to ensure that scHybridNMF did not return the same clusters as k-means. For *α* and *tol*, we recommend using values between 0 and 1.

### 2.5 Convergence of Algorithm

We used a block coordinate descent (BCD) framework to optimize Eq. 3. BCD solves subgroups of problems for a set of variables of interest, which iteratively minimizes the total objective function. We used the minimization version of the two-block BCD method, which assigned $H_{A}^{\left(j\right)}$ and $W_{A}^{\left(j\right)}$ values that minimized Eq. 3 one-at-a-time.

An important theorem regarding BCD states that if a continuously differentiable function over a set of closed convex sets is minimized by BCD, every limit point obtained from uniquely minimizing the subproblems in BCD is a stationary point (Bertsekas et al., 1997). This theorem has the additional property that the uniqueness of the minimum is not necessary for a two-block BCD nonlinear minimization scheme (Grippo and Sciandrone, 2000). This was used to show the convergence of a two-block formulation for solving regular NMF *via* ANLS (Kim et al., 2014).

Given the constrained nonlinear minimization objective in Eq. 3, we rewrote the block coordinate descent as two ANLS formulations, which follow from Eq. 6 and Eq. 7:

$$H_{A}{\left(:,i\right)}^{\left(j\right)}\leftarrow \underset{H_{A}\left(:,i\right)\geq 0}{\arg\;\min}{\left\|\left(\begin{array}{c} W_{A}^{\left(j-1\right)}\\ \sqrt{\alpha }*\mathrm{diag}\left(C\left(:,i\right)\right) \end{array}\right)H_{A}\left(:,i\right)-\left(\begin{array}{c} A\left(:,i\right)\\ 0_{k} \end{array}\right)\right\|}_{F}^{2},$$ (8)

$$W_{A}^{\left(j\right)}\leftarrow \underset{W_{A}\geq 0}{\arg\;\min}{\left\|{\left(H_{A}^{\left(j\right)}\right)}^{T}W_{A}^{T}-A^{T}\right\|}_{F}^{2}.$$ (9)

Eqs. 8 and 9 were executed iteratively to solve for *H*_*A*_ and *W*_*A*_. We considered Eq. 8 to be one block calculation for the entire *H*_*A*_ matrix because the calculation of a column of $H_{A}^{\left(j\right)}$ does not involve any other column. Eqs. 8 and 9 constituted a valid minimization scheme equivalent to minimizing Eq. 3. As such, the theorem by Bertsekas is applicable to this two-block BCD scheme for solving scHybridNMF (Bertsekas et al., 1997; Kim et al., 2014):

*THEOREM 1 Every limit point $\left\{W_{A}^{\left(j\right)},H_{A}^{\left(j\right)}\right\}$ calculated iteratively *via* Eqs. 8–9 is a stationary point of Eq. 3*.

## 3 Results

We tested the performance of scHybridNMF against simulated and real data. For real data, we experimented on the STARmap and seqFISH+ datasets, both of which catalogue the mouse brain cortex (Eng et al., 2019). For STARmap, we compared against sparse NMF and k-means clustering to show an improvement of our hybrid scheme over each method. For the simulated data and seqFISH+, we also compared against HMRF (Zhu et al., 2018), a method that also performs consensus cell clustering on gene expression and cell location data. HMRF models cell locations as nodes on a graph, where cells are connected if they are neighbors in location. It clusters cells by searching for coherent gene expression patterns within neighboring cells.

We implemented the code in MATLAB 2019b. For sparse NMF, we used MATLAB code presented by Kim and Park (Kim and Park, 2008). All experiments were executed on a computer with 2.4 GHz 8-Core Intel Core i9 and 32 GB 2400 MHz DDR4 RAM.

### 3.1 Simulated Data

**FIGURE 1:**
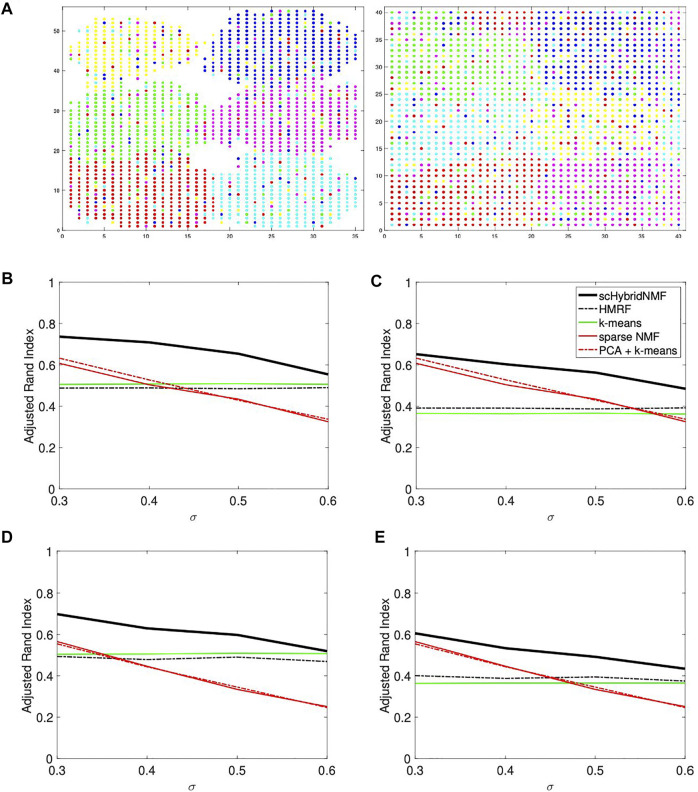
**(A)** An example of noise in location data. The data had *σ* = 0.3 and 20% noise. In each plot, there are six point colors that correspond to true cluster labels. Left: strong spatial patterns; right: weak spatial patterns. Note that certain cell types are not contiguous in the right plot. **(B–E)** Performance vs sigma plots for location data with strong spatial patterns. Each plot shares the same legend as plot **(C)**. **(B)** No sampling, 20*%* noise. **(C)** No sampling, 30*%* noise. **(D)** 50*%* sampling, 20*%* noise. **(E)** 50*%* sampling, 30*%* noise.

We used SymSim to simulate single cell gene expression data, where each cell has one of six cell types (Zhang et al., 2019). Each dataset has 1,600 cells and 600 genes. We developed two types of cell location datasets, where one has strong and the other has weak spatial patterns. For each case, we generated location data with 20 and 30% noise by randomly choosing 20 and 30% of the cells and assigning them to locations outside of their original cell type cluster. Adding noise to the locations made the data more realistic. Figure 1A shows an example of location data with 20% noise.

SymSim has a parameter *σ* that adjusts the within-cluster heterogeneity of gene expression. When *σ* increased, the gene expression-based clusters were less separable, and gene expression-based clustering algorithms were less reliable. We used *σ* = (0.3, 0.4, 0.5, 0.6). For each sigma, 10 gene expression-cell location datasets were generated. For each location matrix, we generated 10 noisy location datasets per noise level.

Many current technologies, especially image-based technologies that pairwise measure the gene expression and spatial locations of single cells, cannot also sequence many genes (Zhu et al., 2018; McKinley et al., 2020). To mimic the limitations of current technology, we additionally created gene-sampled data by randomly sampling 50%, or 300, of the genes from each of the original gene expression datasets.

We compared the quality of clusters determined by gene expression clustering, cell location clustering, and hybrid clustering. The methods we used for gene expression clustering were sparse NMF and PCA plus k-means clustering, which provided a baseline for the performance of sparse NMF. For example, a poor performance from PCA plus k-means clustering justified similarly poor performance of sparse NMF. For location-based clustering, we used k-means clustering. To cluster both data types, we used scHybridNMF and HMRF. HMRF uses a parameter, called beta, which accounts for smoothness. We determined the performance of HMRF as the average performance across 5 values, (0, 20, 40, 60, 80), for beta.

We calculated the adjusted Rand index (ARI) between the calculated and ground truth clusters for each clustering method across each experiment. ARI quantifies how similar two clustering schemes are. If a clustering is very similar to the ground truth clustering, the ARI should be close to 1. We used the sparse NMF and k-means clustering that were used in the steps of Algorithm 1 to calculate their respective ARI values.

#### 3.1.1 Location Data With Strong Spatial Patterns

The location data with strong spatial patterns had significant spatial gaps between clusters (Figure 1A, left plot), and k-means clustering did well separating clusters. For these cases, location clustering played a major role in the multimodal clustering scheme. For *σ* = (0.3, 0.4, 0.5, 0.6), we used *α* = (50, 55, 60, 60) and *tol* = (0.02, 0.02, 0.02, 0.04). We used the same parameters for data with and without gene sampling. We plotted the average ARIs as a function of *σ* in Figures 1B–E. Figures 1B,C show the ARIs for data with no gene sampling, and Figures 1D,E show the ARIs for data with 50% gene sampling.

The plots showed a clear improvement of scHybridNMF over every other method. scHybridNMF followed the same performance trend as gene expression-based clustering across each *σ*. In contrast, HMRF’s performance over every *σ* value was constant. This was highly similar to the performance of location-based clustering, which was often outperformed by gene expression clustering.

#### 3.1.2 Location Data With Weak Spatial Patterns

**FIGURE 2:**
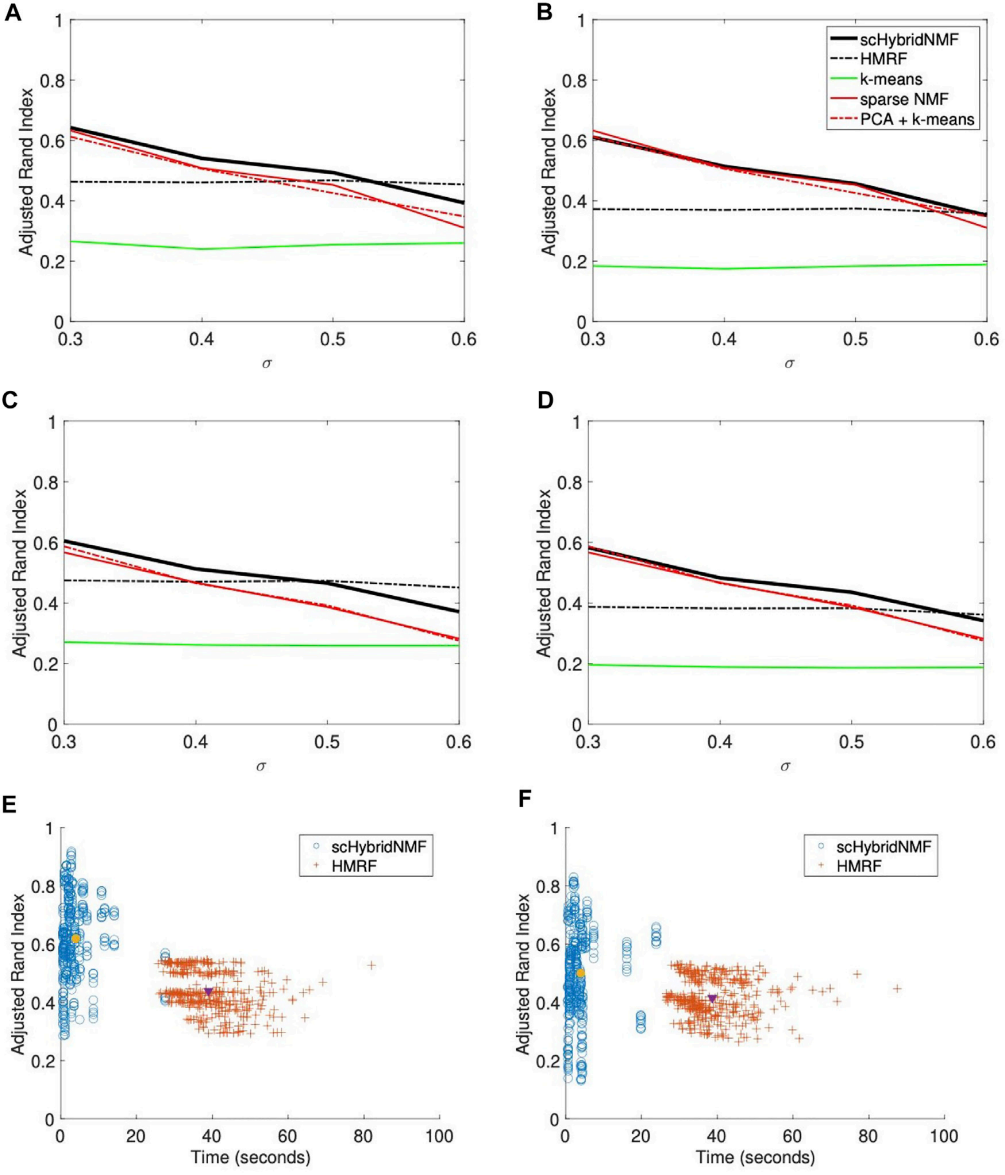
**(A–D)** Performance vs sigma plots for location data with weak spatial patterns. Each plot shares the same legend as plot **(B)**. **(A)** No sampling, 20% noise. **(B)** No sampling, 30% noise. **(C)** 50% sampling, 20% noise. **(D)** 50% sampling, 30% noise. **(E,F)** Time vs performance dot plots of scHybridNMF and HMRF on gene expression data with no gene sampling and location data with strong and weak spatial patterns. **(E)** Strong spatial patterns. **(F)** Weak spatial patterns.

In this location data, the boundaries between clusters were hard to determine (Figure 1A, right plot). As such, k-means clustering experienced more difficulty, and gene expression information was more useful in the multimodal clustering scheme. For *σ* = (0.3, 0.4, 0.5, 0.6), we used *α* = (0.015, 0.02, 0.025, 0.04) and *tol* = (0.2, 0.2, 0.2, 0.2). We used the same parameters for data with and without gene sampling. We plotted the average ARIs as a function of *σ* in Figures 2A–D. Figure 2A,B show the ARIs for data with no gene sampling, and Figures 2C,D show the ARIs for data with 50% gene sampling.

scHybridNMF and HMRF had the same performance trends as they did in Figures 1B–E. However, neither the gene expression nor the cell location data accurately represented the underlying data well—the ARIs and qualities of the gene expression- and location-based clusterings for larger *σ* were very low. Because scHybridNMF drew information from these clusters, it was difficult to gain significantly better information than what was found individually.

scHybridNMF still maintained higher levels of performance in most cases. When *σ* increased, the clusters were less separable with gene expression data, and the performance of sparse NMF decreased. This caused the decrease of the performance of scHybridNMF. Although it did not perform very well with small *σ*, the performance of HMRF was not affected much by the increase of *σ*, and it started to decrease only when *σ* > 0.5. This was likely due to the fact that the neighborhood graph approach used in HMRF is good at learning from location data. However, as evidenced by the performance patterns of HMRF across different *σ* values, HMRF is not able to make full use of high-quality gene expression data.

#### 3.1.3 Timings

We presented two separate dot plots of algorithm completion time vs ARI for each data matrix pair with no gene sampling (Figures 2E,F). An ideal algorithm would have most points in the top-left of the plot; these points correspond to high ARIs with smaller completion times. To show overall trends, we consolidated the noise levels for each plot. For HMRF, we timed from creating the graphical representation to the end for each parameter choice, then averaged the times. For scHybridNMF, we timed from computing sparse NMF to the end. Both algorithm timings matched the values used to compute the ARI values in Figures 1B–E and Figures 2A–D. Figure 2E shows the time and performance data of each point represented in Figures 1B,C, and Figure 2F shows the time and performance data of each point represented in Figures 2A,B.

These experiments showed that scHybridNMF performed well with varying levels of gene sampling and location noise. The fact that scHybridNMF consistently outperformed sparse NMF and k-means indicates that it is likely to be successful on real data.

### 3.2 STARmap Dataset

Wang *et al* developed STARmap, which profiled both “thin” and “thick” cross-sections in the mouse brain cortex (Wang et al., 2018). We used the “thin” dataset, which profiled from layer 1 of the cortex to some of the hippocampus. This dataset has 1,549 cells and 1,020 genes. The cell types noted by Wang et al. (2018) had distinct patterns in their gene expression, cell location, or a combination of both. For example, excitatory neurons may have subtypes specific to certain cortex layers (Tasic et al., 2016). These can be identified by their presence in one or two layers of the cortex, but they are harder to differentiate using only gene expression.

We compared scHybridNMF against sparse NMF and k-means clustering to show that it recovered underlying information that could not be recovered using only one modality of data. We used *k* = 18, which is the same *k* used by Wang et al. (2018). The final clusters we profiled for k-means and sparse NMF were the clusters used as input to scHybridNMF. For scHybridNMF, we set *α* = 0.015 and *tol* = 0.1. This was because the location data was not very separable.

To better compare our clustering results against the underlying cell types, we assigned cell type labels to clusters. We used Scran, a program that detects differentially-expressed (DE) genes given clusters, to find the top 20 such genes per cluster (Lun et al., 2016). We then assigned cell type labels by measuring the overlap of DE genes and marker genes for known cell types in the STARmap data (Wang et al., 2018). The final cluster labels are shown in Supplementary Table S1.

**FIGURE 3:**
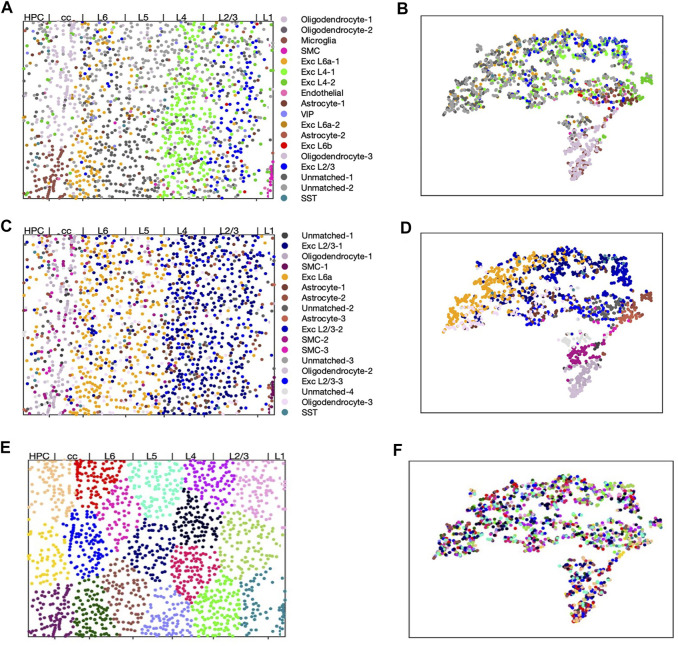
scHybridNMF, sparse NMF and k-means clustering on STARmap data. The layers are labelled by Wang et al. (2018). **(A,C,E)** Visualizing cells in spacial location with cell cluster labels from respectively scHybridNMF, sparse NMF and k-means. **(B,D,F)** cells shown in t-SNE plots of gene expression colored with cluster labels from respectively scHybridNMF, sparse NMF and k-means.

We visualized the clustering results in Figure 3. We first split the different possible cluster colors by the different cell types found, with a particular effort given towards making the excitatory neuron subtype colors distinct. We then consolidated clusters that shared the same cluster label, then assigned them different shades of the color that defined the shared cell type label.

We found that none of the clusters found by k-means clustering matched any known cell types (Figure 3E,F). Using a location-based clustering method only finds clusters based on the location density pattern and the intrinsic characteristics of the clustering method. Therefore, with this STARmap dataset, k-means clustering found similarly-sized and shaped structures that separated the locations evenly. scHybridNMF, on the other hand, found clusters with the striped structures of the layers of the cortex while also recovering cell types that were less spatially conserved (Figure 3A,B).

We performed comprehensive comparison between the results of sparse NMF and scHybridNMF. As a preliminary measure, we computed the ARI between the clusters determined by Wang et al. (2018), noted as ground truth clusters, and the clusters from scHybridNMF and sparse NMF. (Wang et al., 2018). provided labels for 1,389 cells, and we further removed from consideration the cells that Wang et al. (2018) excluded from clustering. This left a total of 1,207 cells for ARI calculation. We found that the ARI between the ground truth and sparse NMF’s clusters to be 0.255, and the ARI between the ground truth and scHybridNMF’s clusters to be 0.21. Sparse NMF’s marginally higher ARI and better-clustered tSNE visualization of gene expression data (Figure 3D) can be explained by the fact that the cluster annotations by Wang et al. (2018) were determined through just the gene expression matrix. However, the spatial distribution of the clusters determined by scHybridNMF better fit the shape of the layer-specific regions in the ground truth labels than the clusters determined by sparse NMF (Figures 3A–D). As such, we further examined both the spatial and gene expression components of the cell type annotations.

Most of the clusters recovered by sparse NMF were similar to those found by scHybridNMF, but scHybridNMF was able to recover major cell types that sparse NMF was not able to (Figures 3A–D). These cell types were separable by gene expression, but were more clearly separated by locations. scHybridNMF was able to recover distinct L2/3, L4, and L6a excitatory neurons, while sparse NMF was not.

#### 3.2.1 scHybridNMF Separates Different Types of Excitatory Neurons

Excitatory neurons have layer-based subtypes (Tasic et al., 2016). These subtypes differ in their locations and gene expression profiles, and each have their own marker genes (Tasic et al., 2016; Wang et al., 2018). Here, we show that scHybridNMF better isolated three subtypes of excitatory neurons, L2/3, L4 and L6a, than sparse NMF.

**FIGURE 4:**
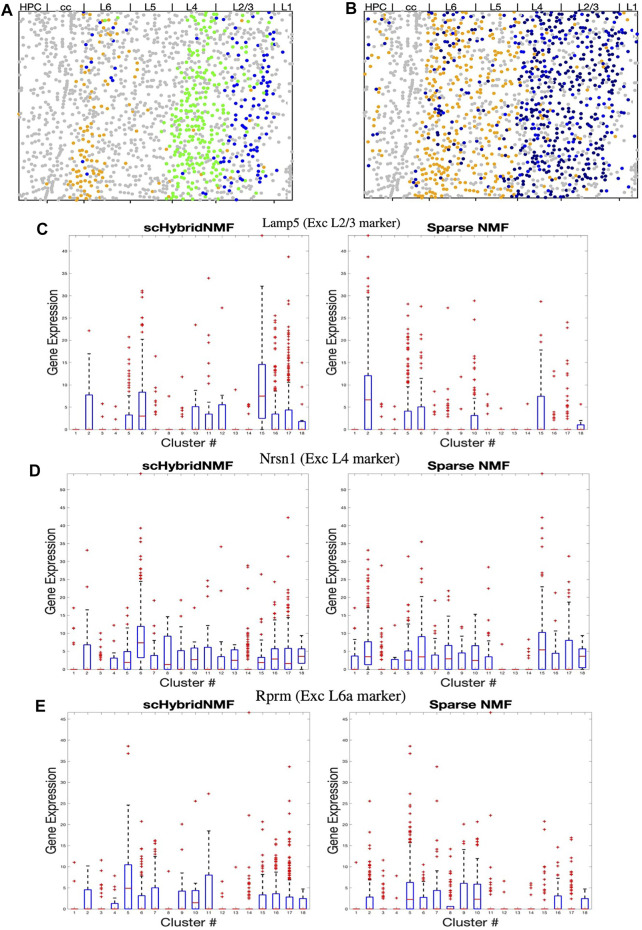
**(A–B)** Dot plots of clusters that best match L2/3, L4, and L6a excitatory neurons. All other clusters are in grey. **(A)** Cluster 5 (L6a, orange), 6 (L4, green), and 15 (L2/3,blue) from scHybridNMF. **(B)** Clusters 5 (L6a, orange), 15 (L2/3, black), and 2 (L2/3, black) from sparse NMF. **(C–E)** Box plots of the expressions of Lamp5, Nrsn1, and Rprm across each cluster.

In Figure 4A,B, we highlighted the clusters relevant to L2/3, L4 and L6a excitatory neurons while keeping other clusters in grey. We observed two separate clusters with scHybridNMF in the upper layers of the brain cortex that corresponded to L2/3 and L4 excitatory neurons (blue and pink clusters in Figure 4A, Supplementary Table S1). In contrast, sparse NMF was not able to detect two clear clusters for L2/3 and L4 excitatory neurons. In fact, there were no cluster found by sparse NMF that could be mapped to L4 excitatory neurons (Supplementary Table S1). Additionally, the clusters that were annotated as L6a excitatory neurons in each method had very different location distributions (Figure 4A,B). Compared to sparse NMF, the cell types annotated by the scHybridNMF clustering were more in line with the layer structure.

We then investigated whether the expression of marker genes supported the clustering by scHybridNMF. We examined Lamp5, Nrsn1, and Rprm, which are noted by (Wang et al., 2018) to be marker genes for L2/3, L4, and L6a excitatory neurons. First, we showed that the expression level of these genes exhibited the spatial pattern of the corresponding layer (Supplementary Figure S1). Then, we compared the differential expression of these genes across scHybridNMF and sparse NMF clusters, shown in box plots in Figures 4C–E.

We used normalized, log-transformed gene expressions to create box plots of the genes across each cluster. Clusters 15 and 6 of scHybridNMF, which were annotated as L2/3 and L4 excitatory neurons, distinctly exhibited higher expressions of Lamp5 and Nrsn1. This differentiation supported the location-based separation of the two excitatory neuron subtypes. On the other hand, for sparse NMF, clusters 2 and 15 had a highly differential level of expression of Lamp5 in Figure 4C. However, the clusters that exhibited high levels of Nrsn1 were also clusters 2 and 15, which were labeled as L2/3 excitatory neurons during the annotation procedure (Figure 4D). The third sparse NMF cluster annotated as L2/3 excitatory neurons, cluster 10, did not exhibit differential expression of these genes (Figure 4C,D).

We additionally observed that scHybridNMF was better able to recover L6a excitatory neurons than sparse NMF. L6a excitatory neurons highly expressed Rprm, were located in the deeper parts of the cortex, and were arranged in a layer-like structure (Supplementary Figure S1). Cluster 5 from both scHybridNMF and sparse NMF corresponded to L6a excitatory neurons (Supplementary Table S1). Cluster 5 of scHybridNMF showed a more distinct expression of Rprm compared to cluster 5 of sparse NMF (Figure 4E). Its spatial pattern, in Figure 4A, also more closely matched the spatial pattern of the cells that highly exhibited Rprm.

It is worth noting that the cell type annotations obtained in Supplementary Table S1 were based on multiple marker genes per cell type. For example, we additionally found that Nrep and Zmat4, noted by (Wang et al., 2018) to be marker genes for L4 excitatory neurons, exhibited the same differential expression for cluster 6 of scHybridNMF. Overall, we showed that scHybridNMF found excitatory neuron subtypes better than sparseNMF in terms of both cell locations and marker gene expression levels.

### 3.3 seqFISH+ Dataset

Eng et al. (2019) profiled the mouse brain cortex and sub-ventricular zone (SVZ) across 7 fields of view (FOV) using the seqFISH+ technique. Five of the FOV were taken from the visual cortex, and 2 from the SVZ. We analyzed 523 cells in the 5 visual cortex FOVs, which encompassed cells from L1 to L6. The gene expression levels of 10,000 genes and locations were profiled for each cell. We computed the means and standard deviations of each gene’s expression levels across each cell, and we kept the genes with means greater than 0.7 and correlations of variation greater than 1.2. This left 1,047 genes. We then added all of the marker genes from Tasic et al. (2016) that were not already in the set of 1,047 genes, which resulted in a total of 1,198 genes.

We set the number of clusters, *k*, to be 19. The labels for the original seqFISH+ dataset were derived from the 49 transcriptomic cell types identified by Tasic et al. (2016). By grouping together cell types in the minor 49, we found 20 cell types. We then explored different numbers of clusters around 20, and found that *k* = 19 gave the most intriguing results. For scHybridNMF, we set *α* = 45 and used a tolerance of 0.05. For the HMRF algorithm, we used a beta value of 10, which was the beta value that gave clusters that were the most consistent with the underlying anatomical structure of the visual cortex.

We used Scran to find the top 20 DE genes per cluster (Lun et al., 2016). We then cross-referenced these with marker genes found by Tasic et al. (2016) and Eng et al. (2019) to map the clusters to tentative cell types. However, certain cell types from Eng et al. (2019) did not match the actual cell locations within the brain cortex. For example, cells annotated as layer 2 excitatory neurons seemed to reside in deeper cortex layers. As such, we considered the location-specific cell type information provided by Tasic et al. (2016) with a higher degree of confidence, and did not compute the ARI with the labels provided by Eng et al. (2019).

**FIGURE 5:**
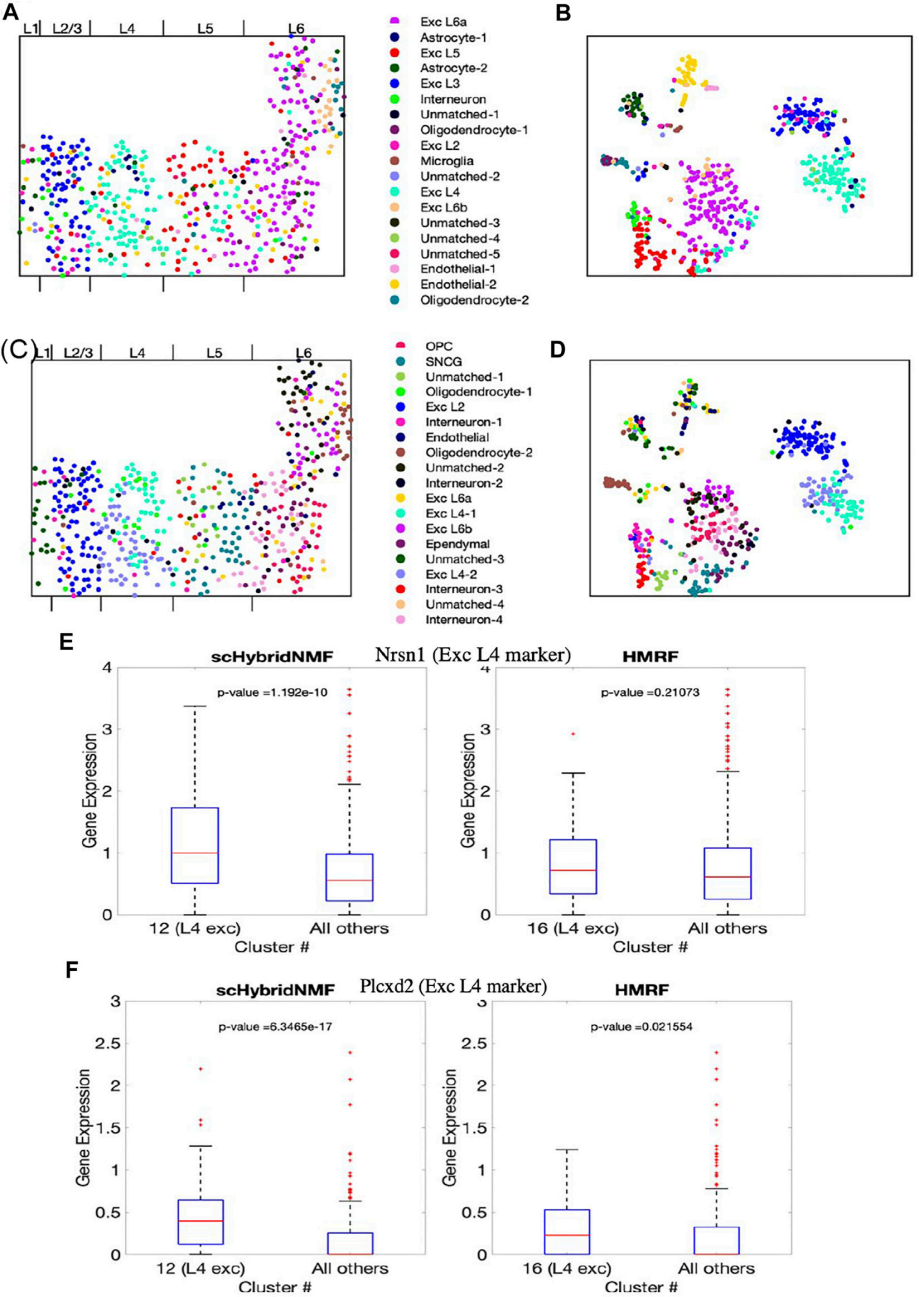
The clustering results of scHybridNMF and HMRF on seqFISH+ data. **(A,C)** Cells visualized in spatial locations with clustering labels from respectively scHybridNMF and HMRF. **(B,D)** Cells visualized in t-SNE plots of gene expression with clustering labels from respectively scHybridNMF and HMRF. The cluster labels are shown in the middle. The layers are labelled by Dries et al. (2021). **(E,F)** Box plots of the expressions of Nrsn1 and Plcxd2 in cells in exc L4 clusters vs all other cells. *p*-values were calculated with a two-sample *t*-test that tested if the population mean of exc L4 clusters were larger than that of the rest of the cells.

The final cluster labels are shown in Supplementary Table S2. We visualized the cluster results of scHybridNMF and HMRF on the cell location and gene expression spaces (Figures 5A–D). We again split the different possible cluster colors by the different labels, with a particular effort given towards making the excitatory neuron subtype colors distinct. We then consolidated clusters that shared the same cluster label, then assigned them different shades of the color that defined the shared cell type label.

As a preliminary reference, we calculated the Silhouette values of the clusterings found by scHybridNMF and HMRF for gene expression values. However, both methods had very similar performances across every cluster found, even clusters that were left unmapped. As such, we conducted a gene ontology (GO) term analysis for the DE genes found by Scran.

#### 3.3.1 scHybridNMF Detects L4 Excitatory Neurons

Layer-specific excitatory neurons form contiguous, column-like structures, and they also have unique gene expression profiles (Tasic et al., 2016). The Giotto authors labelled distinct physical layers, numbered 1, 2/3, 4, 5, and 6, in the seqFISH+ dataset (Dries et al., 2021). We found that there were excitatory neuron subtypes that generally corresponded to each of layers 2/3 to 6. In particular, we found that scHybridNMF was able to recover a cluster (cluster 12 in Supplementary Table S2) that better corresponded to L4 excitatory neurons than HMRF’s cluster (cluster 16 in Supplementary Table S2).

To further investigate this, we looked into the expressions of marker genes, especially Nrsn1 and Plcxd2. Nrsn1 was noted by Eng et al. (2019) to be a marker gene for excitatory neurons, and is visibly highly expressed in layer 4 of the cortex. Plcxd2 is shown by (Wang et al., 2018) to be a marker gene for neuronal cells, especially L4 and L5 excitatory neurons, but we show that in the seqFISH+ dataset, this is uniquely highly expressed in layer 4. All other marker genes are shown in Supplementary Figures S2,S3.

First, we saw that the cells that highly expressed these genes were grouped together in a layer-like shape (Supplementary Figures S2A,B), confirming the marker genes’ spatial patterns. We then visualized the different marker gene expressions with box plots, comparing the expressions within L4 excitatory neuron clusters of scHybridNMF and HMRF against the rest of the cells (Figures 5E,F). We found that, with a threshold of *p* < 0.01, cluster 12 of scHybridNMF exhibited a significantly higher expression of Nrsn1 and Plcxd2 than the rest of the cells (Figures 5E,F). In contrast, HMRF failed to reject the null hypothesis, with *p*-values of 0.21 and 0.02.

#### 3.3.2 Layer 6b Excitatory Neurons

The deepest layers of the mouse brain cortex are L5 and L6, where L6 can further be split into L6a and L6b. L6b exhibits both a distinct location and gene expression profile from L6a, which tends to be closer to L5. Using scHybridNMF, we found that the seqFISH+ dataset showed clear location- and gene expression-based evidence for a distinct L6b excitatory neuron cell type. Tasic et al. (2016) give marker genes for L6a and 6b excitatory neurons, which are Rprm and Ctgf, respectively. In the seqFISH+ dataset, these exhibited strong spatial coherency, where we observed a clear boundary between cells that highly express Rprm vs Ctgf (Supplementary Figure S4), which clearly divided the two types of L6 excitatory neurons.

**FIGURE 6:**
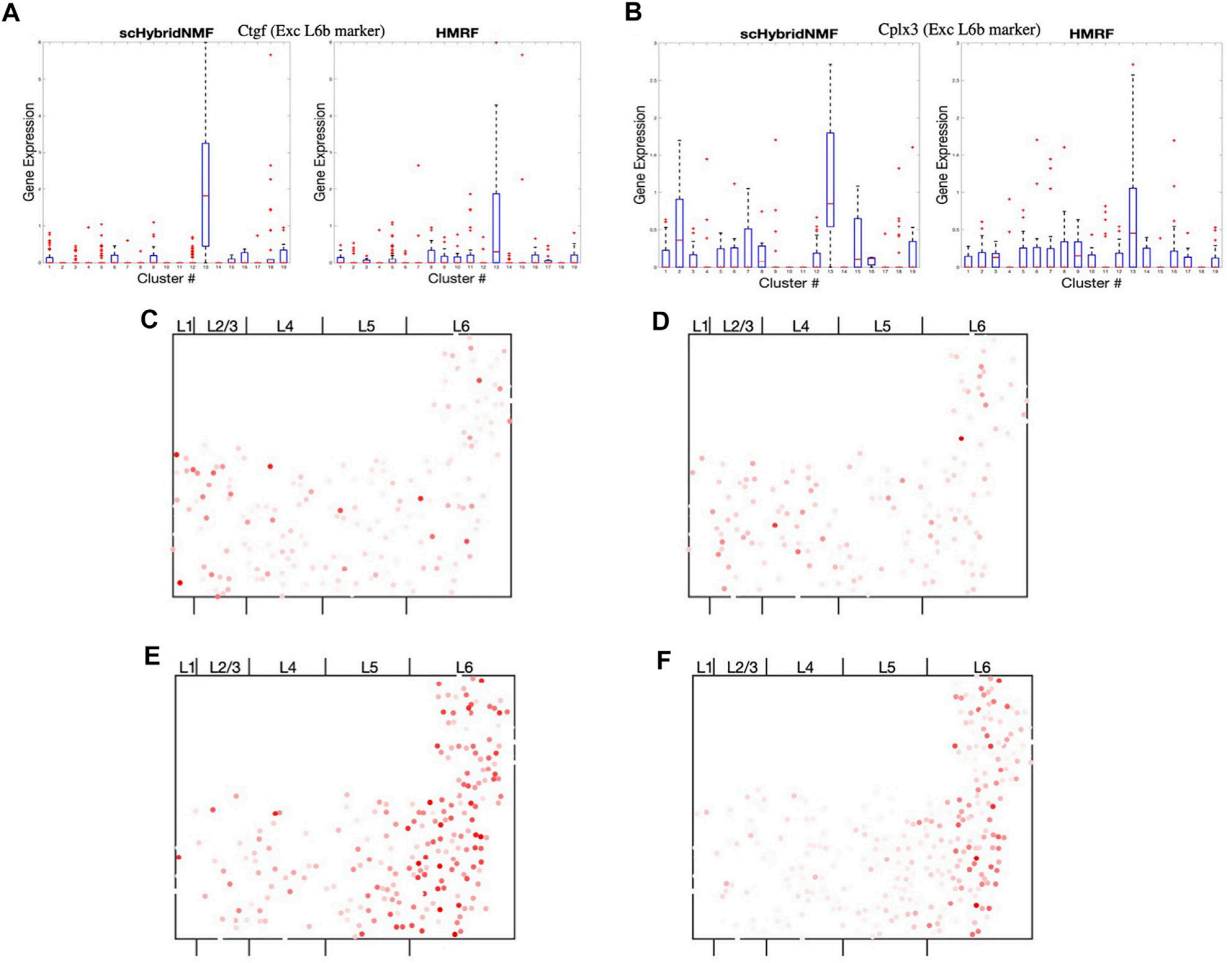
**(A,B)** Box plots of the expressions of Ctgf and Cplx3 across each cell, grouped by cluster. **(C,D)** Dot plots of the expressions of Cpne2 and Ptgfr across each cell. These genes are marker genes of L5a and L5b excitatory neurons (Tasic et al., 2016). **(C)** Cpne2 (L5a marker). **(D)** Ptgfr (L5b marker). **(E,F)** Dot plots of the expressions of Islr2 and Rprm across each cell. We propose Islr2, a DE gene recovered through Scran on scHybridNMF clusters, as a marker gene for L6 excitatory neurons. We show the expression of Rprm as a baseline. **(E)** Islr2 (scHybridNMF). **(F)** Rprm (L6a, Tasic, *et al*).

scHybridNMF was able to recover L6b excitatory neurons better than HMRF. To measure the differential gene expression across each cluster found by HMRF and scHybridNMF, we measured the expression of Ctgf and Cplx3, marker genes cited by Tasic et al. (2016), in Figures 6A,B. Because both genes were markers for L6b excitatory neurons, high-quality clusters are expected to exhibit a strongly distinct level of expression for these genes. We used the normalized, log-transformed gene expressions to create box plots of the expression statistics across each cluster. The side-by-side analysis of the two algorithms showed that the L6b cluster found by scHybridNMF exhibits a more distinct pattern of gene expression than the L6b cluster found by HMRF.

The region of cells highly expressing Ctgf in Supplementary Figure S4 was small and sliver-like, and it bordered the rightmost side of layer 6. We found that the spatial location of the L6b cluster from scHybridNMF seemed to align more closely to this shape than the cluster from HMRF (Supplementary Figure S5). The cluster from HMRF included cells that were part of L6a.

#### 3.3.3 scHybridNMF Refines Marker Gene Lists

##### Reducing False Positives of Layer 5 Excitatory Neuron Markers

The marker gene lists noted by Tasic et al. (2016) and by Dries et al. (2021) provided a basis for cell type annotations and interpretations of results in subsequent research. However, the markers obtained in Tasic et al. (2016) were based on scRNA-seq data only, and some of the location-specific marker genes may not actually demonstrate the expected location pattern. Indeed, from the DE analysis based on the clusters obtained by scHybridNMF, we found there were certain marker genes noted by Tasic et al. (2016) that did not exist in the DE results. We focused on the marker genes for L5 excitatory neurons and further investigated the spatial pattern of these genes.

Tasic et al. (2016) catalogued 3 separate excitatory neuron types corresponding to L5. They were L5, L5a, and L5b excitatory neurons, where L5a and L5b distinguish the shallower and deeper regions of L5, respectively. The L5 excitatory neuron type referenced the entirety of layer 5. Of the 10,000 genes measured in seqFISH+, we found 17 were labeled as marker genes for only L5, L5a, or L5b excitatory neurons in Tasic et al. (2016). However, none of these genes exhibited any particular spatial pattern associated with L5. Examples of the spatial patterns are given in Figures 6C,D and Supplementary Figure S6.

##### Potential New Marker Gene for L6a Excitatory Neurons

Cluster 1 of scHybridNMF was annotated as L6a excitatory neurons both by gene expression and cell locations (Supplementary Table S2). Rprm is a marker gene from Tasic et al. (2016), and it exhibited a strong, spatially-conserved pattern in the seqFISH+ data (Figure 6F. We found another gene, Islr2, as a potential marker gene for L6a excitatory neurons. This is because it was differentially-expressed in cluster 1 [through Scran (Lun et al., 2016)], exhibited strong spatial cohesiveness, and was involved in neuron function and development (Abudureyimu et al., 2018) (Figure 6E). It was also found to be spatially concentrated in L5/6 by Giotto (Dries et al., 2021).

## 4 Conclusion and Discussion

We presented a hybrid clustering approach that can better identify cell types by incorporating sparse NMF and k-means clustering, which work well on high-dimensional gene expression and low-dimensional location data. We demonstrated the robustness of scHybridNMF through experiments on both simulated and real data.

We showed that the hybrid framework was particularly useful when the performance of sparse NMF was affected by a low number of genes profiled or high within-cluster heterogeneity. scHybridNMF also outperformed k-means clustering under realistic scenarios. Through combining two classical methods for clustering, sparse NMF and k-means, scHybridNMF made better use of both data than either of the standalone methods as well as an existing method HMRF.

We also observed that scHybridNMF found biologically-meaningful clusters within real data. We analyzed the biological relevance of the clusters using cluster-specific DE genes that were found using cell cluster membership information. However, similar metagene analysis can be done using *W*_*A*_, the cluster representative matrix. This matrix, which contains the final gene expression representatives of each cluster, was built using cell location and gene expression information. As such, *W*_*A*_ is constructed in such a way that incorporates both sources of information, and analyzing the differential expression of genes across different cluster representatives is intuitive. Each row of *W*_*A*_ corresponds to each gene, and the more variation of values there is in a row, the more likely the corresponding gene is biologically meaningful for cell type identification.

scHybridNMF is inherently flexible, owing to its matrix low-rank approximation formulation. As such, it can be extended via additional matrix terms and constraints to include more types of data or to perform biclustering. For example, we can include potential gene-gene interaction data to perform co-clustering of both cells and genes. The inferred gene clusters can be further used to study regulatory mechanisms in the cells and reconstruct gene regulatory networks.

## Data Availability

scHybridNMF is available at github.com/soobleck/scHybridNMF. The simulated data and processed real data used in this study are also in the same GitHub repository. Further inquiries can be directed to the corresponding authors.
