# Supplementary material for: Hybrid Clustering of Single-Cell Gene Expression and Spatial Information via Integrated NMF and K-Means
Source: Front Genet. 2021 Nov 8;12:763263. doi: 10.3389/fgene.2021.763263 (PMC8606648; doi:10.3389/fgene.2021.763263)
Supplement: Supplementary file 1 [file DataSheet1.PDF]

Hybrid clustering of single-cell gene expression and cell spatial information via integrated NMF and k-means

Supplementary Materials

Sooyoun Oh, Haesun Park, Xiuwei Zhang

Table of contents

Table of contents 1

Supplementary Figures 2

Supplementary Tables 8

Supplementary Figures

Supplementary Figure 1: Spatial distribution of Lamp5 (Exc L2/3 marker), Nrsn1 (Exc L4 marker) and Rprm (L6a marker) in STARmap. The intensity of the colors of the dots depend on the relative expression of the gene in comparison to the rest of the cells. If a cell exhibited multiple marker genes, the colors of each of the markers were mixed in the corresponding dot. A lighter colored dot indicates a relatively smaller expression level of the marker genes.

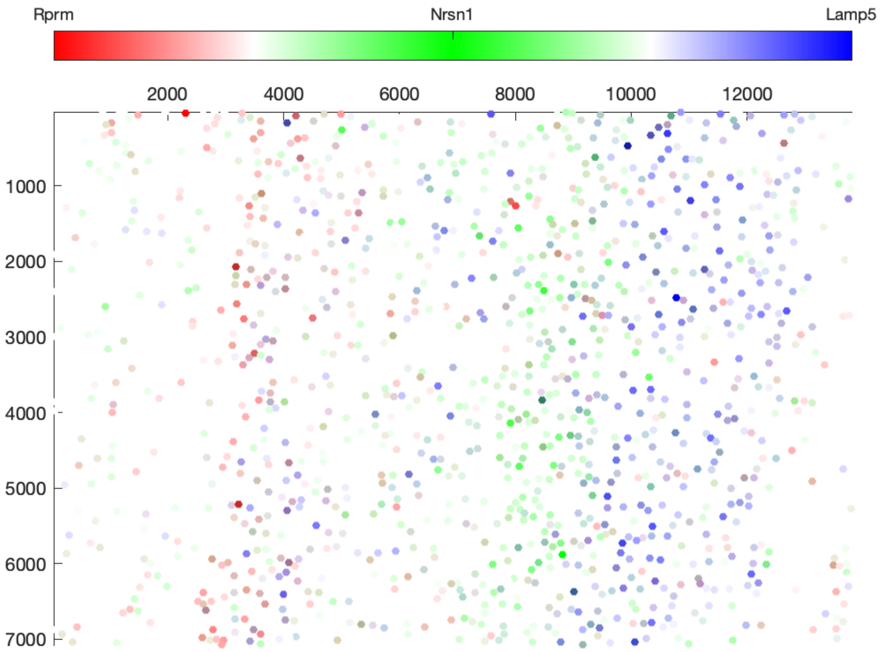

Supplementary Figure 2: Spatial distribution of six L4 excitatory neuron marker genes in seqFISH+. These are listed as marker genes by Eng, *et al.* (2019) and Wang, *et al.* (2018). The intensity of the dot colors depend on the relative expression of the gene in comparison to the rest of the cells. Other marker genes for L4 excitatory neurons not pictured: Abhd8, Atp1a1, Car10, Grm2, Lingo1, Ngef, Nptxr, Nr1d1, Nr2f1, Nr4a1, Pgbd5, Prmt8, Rtn4r, Rusc1, Stx1a, Syngap1, Synpo.

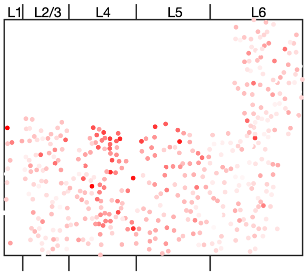

(a) Nrsn1 (Eng, *et al.*)

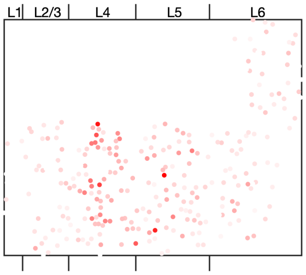

(b) Plcxd2 (Wang, *et al.*)

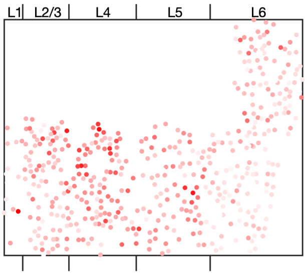

(c) Actr1b (Eng, *et al.*)

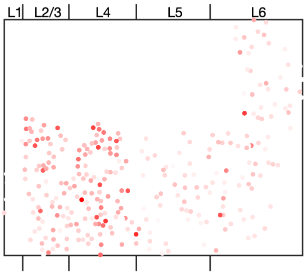

(d) Dact2 (Eng, *et al.*)

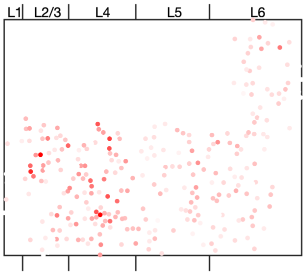

(f) Osbp2 (Eng, *et al.*)

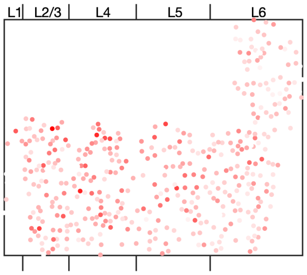

(g) Svop (Eng, *et al.*)

Supplementary Figure 3: Box plots of the expressions of marker genes (from Fig. S2) in cells in exc L4 clusters vs all other cells. P-values were calculated with a two-sample t-test that tested if the population mean of exc L4 clusters were larger than that of the rest of the cells. Other marker genes for L4 excitatory neurons not pictured: Abhd8, Atp1a1, Car10, Grm2, Lingo1, Ngef, Nptxr, Nr1d1, Nr2f1, Nr4a1, Pgbd5, Prmt8, Rtn4r, Rusc1, Stx1a, Syngap1, Synpo.

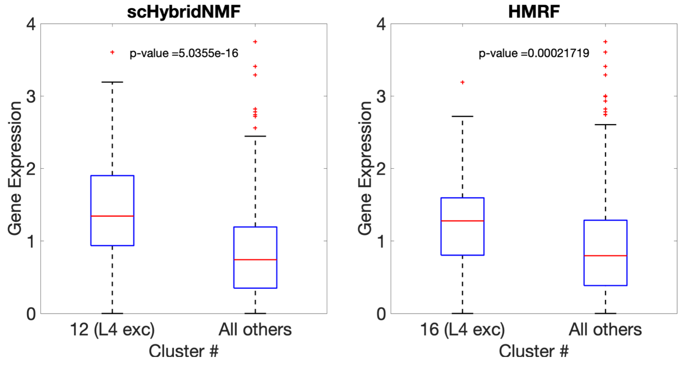

(a) Actr1b

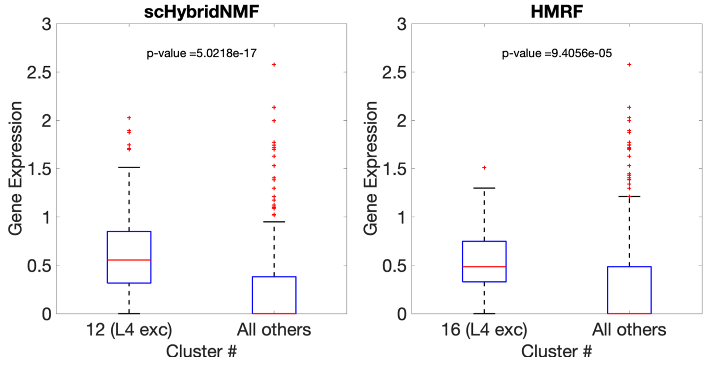

(b) Dact2

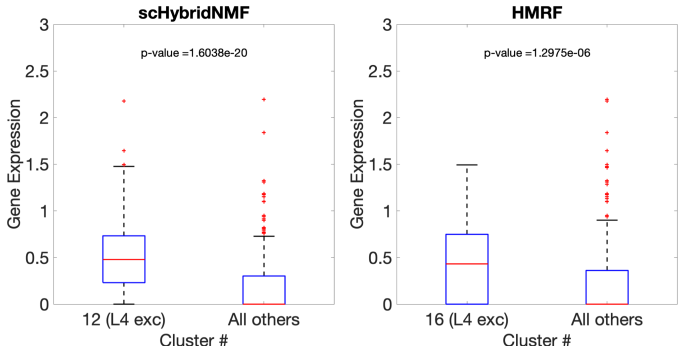

(c) Osbp2

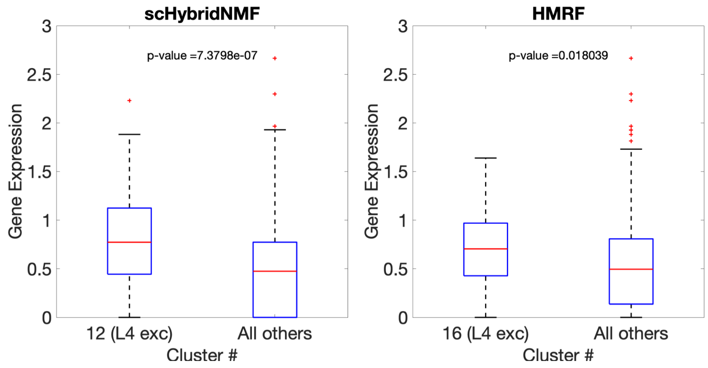

(d) Svop

Supplementary Figure 4: Spatial distribution of Rprm (Exc L6a marker) and Ctgf (Exc L6b marker) in seqFISH+. Both are listed as marker genes by Tasic, *et al.* (2016). The intensity of the colors of the dots depend on the relative expression of the gene in comparison to the rest of the cells. If a cell exhibited multiple marker genes, the colors of each of the markers were mixed in the corresponding dot. A lighter colored dot indicates a relatively smaller expression level of the marker genes.

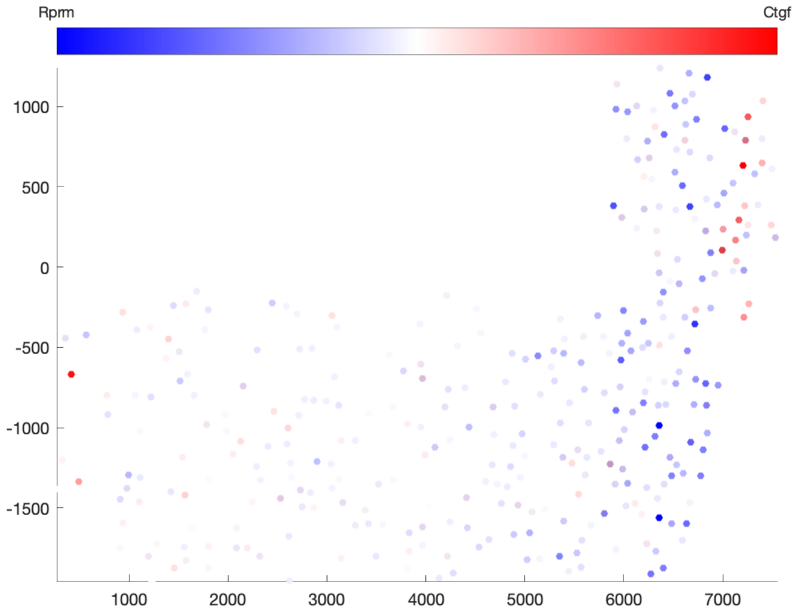

Supplementary Figure 5: L6b clusters from HMRF and scHybridNMF in seqFISH+.

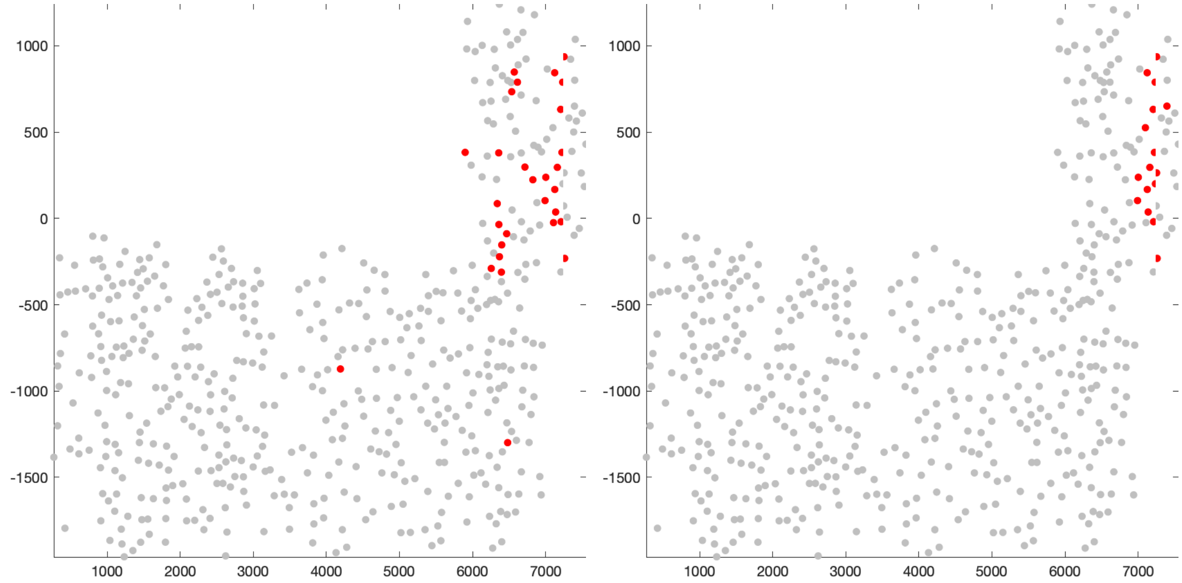

(a) HMRF

(b) scHybridNMF

Supplementary Figure 6: Spatial distribution of all marker genes for L5 excitatory neurons by Tasic, *et al.* (2016). The intensity of the colors of the dots depend on the relative expression of the gene in comparison to the rest of the cells.

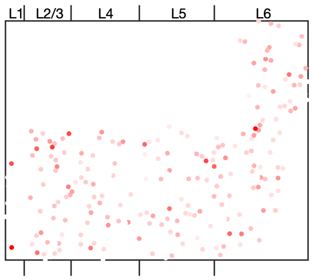

(a) Marker genes for L5 excitatory neurons by Tasic, *et al.* (2016). (left-right) Hhatl, Itga7.

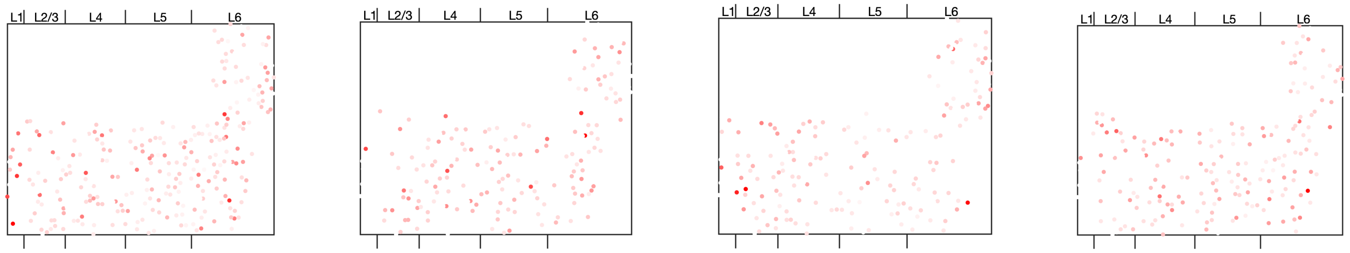

(b) Marker genes for L5a excitatory neurons by Tasic, *et al.* (2016). (left-right) Aldh1l1, Deptor, Foxo1, Pacsin2.

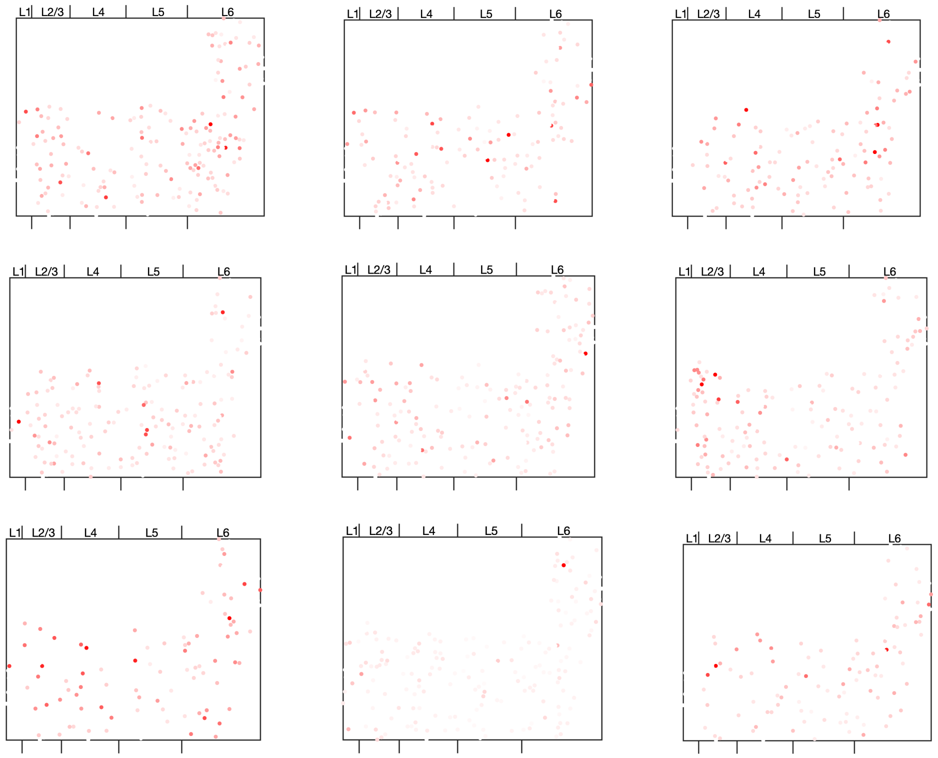

(c) Marker genes for L5b excitatory neurons by Tasic, *et al.* (2016). (left-right, top-bottom) Col6a1, Ddit4l, Fam84b, Kctd8, Mc4r, Ngb, Scml2, Sema3c, Stac.

Supplementary Tables

Supplementary Table 1: Cell type label mappings per cluster based on marker genes from Wang, et al. (2018), cell locations, or both. Annotations based on both are in italics. Clusters were labeled as “Unmatched” if there were fewer than 5 cells in the cluster, or if the marker gene annotation indicated that the cluster corresponded to a cell type that exhibited a spatial structure that did not match the structure of the cluster.

#	Sparse NMF	Match Type	scHybridNMF	Match Type
1	Unmatched		Oligodendrocyte	Marker gene
2	Exc L2/3	Cell location	Oligodendrocyte	Marker gene
3	Oligodendrocyte	Marker gene	Microglia	Marker gene
4	SMC	Marker gene	SMC	Marker gene
5	Exc L6a	Cell location	*Exc L6a*	Both
6	Astrocyte	Marker gene	*Exc L4*	Both
7	Astrocyte	Marker gene	Exc L4	Marker gene
8	Unmatched		Endothelial	Marker gene
9	Astrocyte	Marker gene	Astrocyte	Marker gene
10	Exc L2/3	Marker gene	VIP	Marker gene
11	SMC	Marker gene	Exc L6a	Marker gene
12	SMC	Marker gene	Astrocyte	Marker gene
13	Unmatched		Exc L6b	Marker gene
14	Oligodendrocyte	Marker gene	Oligodendrocyte	Marker gene
15	Exc L2/3	Marker gene	*Exc L2/3*	Both
16	Unmatched		Unmatched	
17	Oligodendrocyte	Marker gene	Unmatched	
18	SST	Marker gene	SST	Marker gene

Supplementary Table 2: Cell type label mappings per cluster based on marker genes from Tasic, *et al.* (2016) and Eng, *et al.* (2019), cell locations, or both. Annotations based on both are in italics. If there are competing annotations from both marker gene lists, we defaulted to Eng, *et al.* (2019). Clusters were labeled as "Unmatched" if there were fewer than 5 cells in the cluster, or if the marker gene annotation indicated that the cluster corresponded to a cell type that exhibited a spatial structure that did not match the structure of the cluster.

#	HMRF	Match Type	scHybridNMF	Match Type
1	OPC	Marker gene	*Exc L6a*	Both
2	SNCG	Marker gene	Astrocyte	Marker gene
3	Unmatched		Exc L5	Cell location
4	Oligodendrocyte	Marker gene	Astrocyte	Marker gene
5	*Exc L2*	Both	*Exc L3*	Both
6	Interneuron	Marker gene	Interneuron	Marker gene
7	Endothelial	Marker gene	Unmatched	
8	Oligodendrocyte	Marker gene	Oligodendrocyte	Marker gene
9	Unmatched		Exc L2	Cell location
10	Interneuron	Marker gene	Microglia	Marker gene
11	*Exc L6a*	Both	Unmatched	
12	Unmatched		*Exc L4*	Both
13	Exc L6b	Marker gene	*Exc L6b*	Both
14	Ependymal	Marker gene	Unmatched	
15	Unmatched		Unmatched	
16	*Exc L4*	Both	Unmatched	
17	Interneuron	Marker gene	Endothelial	Marker gene
18	Unmatched		Endothelial	Marker gene
19	Interneuron	Marker gene	Oligodendrocyte	Marker gene
